# Supplementary material for: Experience and Awareness of Health Managers, Administrators, and Workers on a Hearing Conservation Program in Korea: A Qualitative Study
Source: Int J Environ Res Public Health. 2020 Mar 29;17(7):2302. doi: 10.3390/ijerph17072302 (PMC7177902; doi:10.3390/ijerph17072302)
Supplement: Supplementary file 1 [file ijerph-17-02302-s001.pdf]

FGD & IDI guidelines for the hearing conservation program experience and perception evaluation

Ice breaking (10-minute)

• Introduction and purpose explanation on the discussion

• Introduction on the discussion process

- Recording notification and approval confirmation / Inform the discussion process and precautions / Inform about personal information protection

• Participation introduction

Experience of the hearing conserving program (30-minute)

1. Utilization experience of the hearing conserving program

1) "How important do you think the hearing loss problem is?"

▶ Inquiry question

- "How important do you think of the problem compared to the other problems in terms of the industrial health?"
- Review statistics of the hearing loss

2) "How much do you think the hearing loss problem in this country has been publicized?"

▶ Inquiry question

"If not, what do you think is reason behind it?"

Perception of the hearing conserving program (70-minute)

2. Perception of the hearing conserving program

1) "Have you had any experience in applying the hearing conserving program?"

▶ Inquiry question

- If so, "Please describe your experience in detail."
- If not, "Then have you heard of such program? How do you know?"

2) "Do you think it is necessary to have a hearing conserving program?"

▶ Inquiry question

- If not, "what made you think so?"

* Provide introduction material on the hearing conserving program
- The hearing conserving program (HCP) for industrial fields is a noise-induced deafness preventative program for field workers who are exposed to excessive noise.
- The hearing conserving program is constituted in total 6 components; 1) noise measurement; 2) engineering

control of noise and administrative management; 3) wearing hearing protective devices; 4) hearing test and medical judgement; 5) health education and training; 6) record documentation and program effect evaluation.

3) “The hearing conserving program is constituted with the followings. Please take a closer look at each components.”

▶ Inquiry question

- Comprehend problems and improvement plan for each components

4) “What do you think is the reason for reluctant implementation of the hearing conserving program?”

▶ Inquiry question

- Additional confirmation on various challenges in the hearing conserving program

5) “What measures should be made to vitalize the hearing conserving program?”

Discussion wrap up (10-minute)

• Additional and related questions on the discussion

• Other questions

• Conclude the discussion and show gratitude for the participation
